# Interleukin 12: still a promising candidate for tumor immunotherapy?

**DOI:** 10.1007/s00262-014-1523-1

**Published:** 2014-02-11

**Authors:** Witold Lasek, Radosław Zagożdżon, Marek Jakobisiak

**Affiliations:** Department of Immunology, Centre of Biostructure Research, Medical University of Warsaw, Banacha 1a, “F” Bldg, 02-097 Warsaw, Poland

**Keywords:** Interleukin 12, Cancer immunotherapy, Gene therapy, Cytokine

## Abstract

Interleukin 12 (IL-12) seemed to represent the ideal candidate for tumor immunotherapy, due to its ability to activate both innate (NK cells) and adaptive (cytotoxic T lymphocytes) immunities. However, despite encouraging results in animal models, very modest antitumor effects of IL-12 in early clinical trials, often accompanied by unacceptable levels of adverse events, markedly dampened  hopes of the successful use of this cytokine in cancer patients. Recently, several clinical studies have been initiated in which IL-12 is applied as an adjuvant in cancer vaccines, in gene therapy including locoregional injections of IL-12 plasmid and in the form of tumor-targeting immunocytokines (IL-12 fused to monoclonal antibodies). The near future will show whether this renewed interest in the use of IL-12 in oncology will result in meaningful therapeutic effects in a select group of cancer patients.

## Introduction

Interleukin 12 (IL-12) is a pleiotropic cytokine, the actions of which create an interconnection between the innate and adaptive immunity. IL-12 was first described as a factor secreted from PMA-induced EBV-transformed B-cell lines. Based on its actions, IL-12 was initially designated as “cytotoxic lymphocyte maturation factor” [1] and “natural killer cell stimulatory factor” [2]. Due to bridging the innate and adaptive immunity and potently stimulating the production of IFN-γ—a cytokine coordinating natural mechanisms of anticancer defense [3]—IL-12 seemed ideal candidate for tumor immunotherapy in humans. However, severe side effects associated with systemic administration of IL-12 in clinical investigations and the very narrow therapeutic index of this cytokine markedly tempered enthusiasm for the use of this cytokine in cancer patients. Despite those setbacks, IL-12 continues to be the focus of interest in clinical oncology. The present review summarizes the most promising IL-12-based approaches in animal models and discusses clinical trials with special emphasis on ongoing studies aimed at the improvement of the therapeutic efficacy of IL-12 and limitation of its toxicity.

## Biological effects of IL-12 and its role in the antitumor defense mechanisms

The main source of IL-12 in humans is the activated antigen-presenting cells, such as dendritic cells [4], especially of the CD1c^+^ phenotype [5], as well as the hematopoietic phagocytes (monocytes, macrophages, and also neutrophils) [6], but IL-12 can also be produced by other cell types [7, 8]. While IL-12 acts on a variety of immune cells, the overall physiological role for IL-12 seems to be orchestrating the Th1-type immune response against certain pathogens. Also, a range of immunoregulatory activities of IL-12 have raised a profound interest in this cytokine as a potential anticancer agent [9].

### Structure of IL-12 and its cognate receptor

Structurally, IL-12 belongs to type I cytokines and has a four α-helical bundle structure. IL-12 acts in a form of a heterodimeric protein (IL-12-p70; IL-12-p35/p40) consisting of two covalently linked p35 and p40 subunits. Contrary to the heterodimeric form, IL-12-p40/p40 homodimer acts mostly as a competitive suppressant of IL-12-p70 actions [10]. Following the discovery of IL-12, three other members (IL-23, IL-27, and IL-35) have been added to the IL-12 family and shown to play critical roles in Th1 cell functions (reviewed in [11]).

IL-12 is a ligand of a receptor composed of two amino acid chains, IL-12R-β1 and IL-12R-β2. IL-12 receptor is expressed in a constitutive (e.g., IL-12R-β1 in B cells [12]) or inducible (IL-12R-β2 [12]) manner in a variety of immune cells, including NK cells, T, and B lymphocytes. Ligand-bound IL-12R-β2 becomes phosphorylated on tyrosines, which provides harboring sites for two kinases, JAK2 and TYK2. Among the STAT family of transcription factors, STAT4 is considered to be the most specific mediator of cellular responses elicited by IL-12 [13].

### Regulation of IL-12 expression

During the immune response against pathogens, production of an active IL-12-p70 heterodimer can be increased by two types of stimuli: priming and amplification [14]. The priming event is usually mediated via “danger signaling” routes of the immune system, many of them transduced through the toll-like receptor (TLR) family. In macrophages, for instance, IL-12 can be induced following TLR4 ligand—lipopolysaccharide (LPS), and TLR7/8 ligand—R848, binding to their cognate receptors [15]. The amplification signaling is provided by a cytokine network (e.g., by IL-1β [16]) or direct cell–cell contact with other immune cells (e.g., CD40L–CD40 interaction [17]). It is uncertain, however, what exact molecular events underlie triggering the cancer-induced IL-12 production. The most likely mechanism is the CD40L–CD40 interaction [18].

Suppression of IL-12 production is mediated by such cytokines as type I IFNs [19], IL-10, and TGF-β [20] as well as by prostaglandin E_2_ (PGE_2_) that is produced by various cancers [21]. Another suppressive molecule is T-cell immunoglobulin and mucin domain-containing protein 3 (Tim-3), which can inhibit the production of IL-12 by dendritic cells [15] within a tumor environment (highlighted in [22]). Direct cell–cell contact has also been described as a mechanism of decreasing IL-12 production, for instance by tumor-derived CD4^+^CD25^+^ T regulatory (Treg) lymphocytes via CTLA-4-mediated signaling [23] or by CD200–CD200R interactions [24].

### Biological activities of IL-12 as an antitumor cytokine

Although potent antitumor effects of IL-12 are very well established, this cytokine is considered to be incapable of directly inhibiting the cancer growth, although exceptions can occur [25]. Rather, IL-12 acts as a major orchestrator of Th1-type immune response against cancer. Another important notion is that IL-12 appears to elicit more potent antitumor responses when existent directly in the tumor whereabouts, rather than present systemically. In the latter case, especially in humans, toxicities of IL-12 administration seem to prevail over its antitumor effectiveness.

**Fig. 1:**
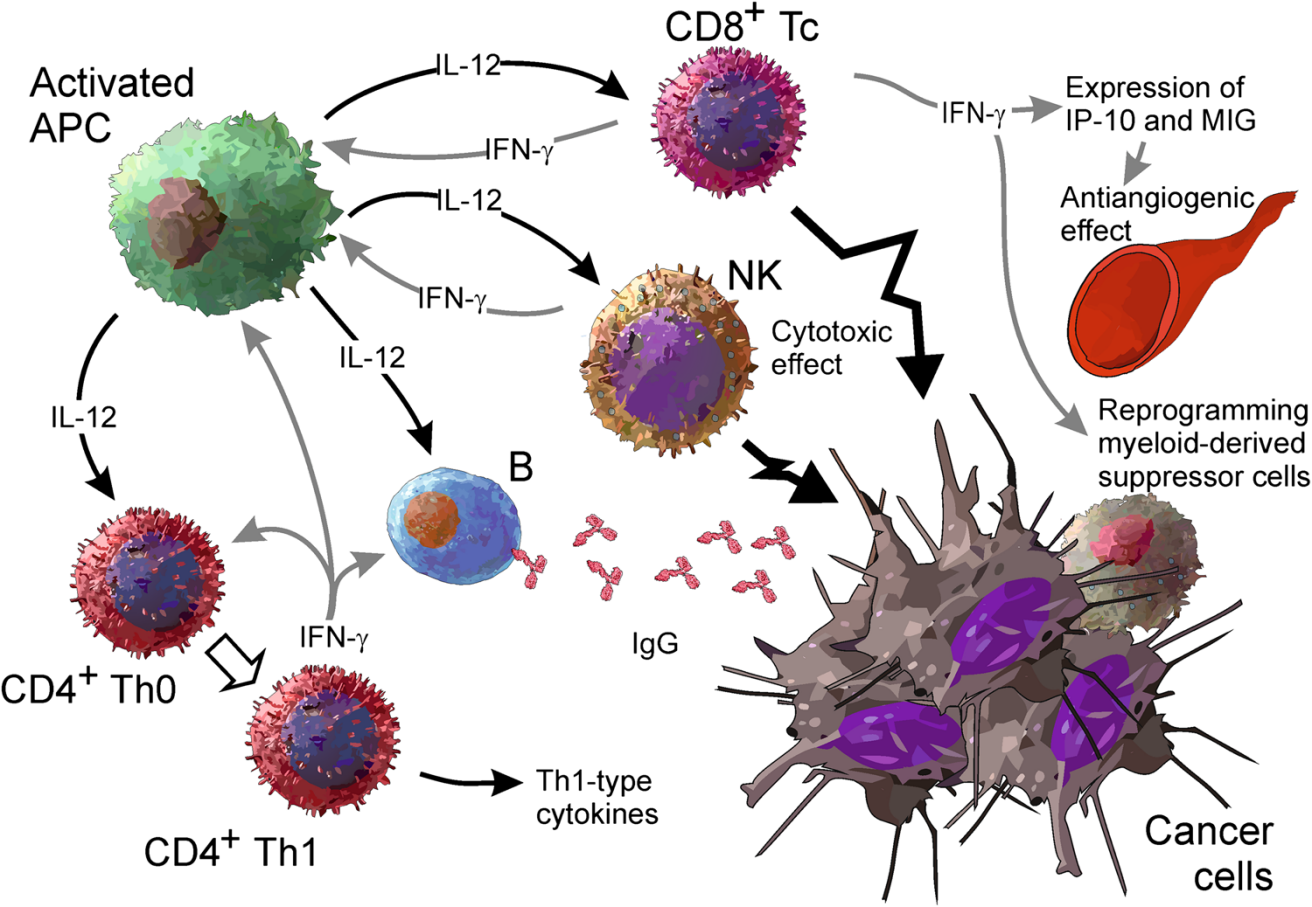
An overview of the biological properties of IL-12 contributing to the antitumor activity of this cytokine (see the text for details). *APC* antigen-presenting cell, *NK* natural killer cell, *Tc* cytotoxic T lymphocyte, *Th* T helper lymphocyte, *IP*-*10* interferon-inducible protein 10, *MIG* monokine induced by interferon γ

The main elements of IL-12 actions are as follows (Fig. 1): increasing production of IFN-γ, which is the most potent mediator of IL-12 actions, from NK and T cells [26]; stimulation of growth and cytotoxicity of activated NK cells, CD8^+^ and CD4^+^ T cells [27], shifting differentiation of CD4^+^ Th0 cells toward the Th1 phenotype [28]; enhancement of antibody-dependent cellular cytotoxicity (ADCC) against tumor cells [29, 30]; and the induction of IgG and suppression of IgE production from B cells [31]. Several other mechanisms, however, also strongly contribute to antitumor activities of IL-12. These are potent antiangiogenic effects via induction of antiangiogenic cytokine and chemokine production [32], remodeling of the peritumoral extracellular matrix and tumor stroma [33], reprogramming of myeloid-derived suppressor cells [34], and changes in processing and increasing expression of MHC class I molecules [35]. All the above mechanisms converge during response against tumors and are postulated to be responsible for the high potency of antitumor effects of IL-12.

The observations on activating the effects of IL-12 on T and NK cells have been made early in the studies on this cytokine and have been a subject of several systematic reviews [36, 37]. A recent study, however, has demonstrated that prolonged treatment with IL-12 can have some detrimental effects on antitumor activity of T cells, by the induction of expression of Tim-3 molecule in T cells [38]. This mechanism is most likely a negative feedback loop preventing the overactivation of the immune system in course of the pathogen invasion, but in the case of a chronic disease like cancer or some infectious diseases [39] can be hampering the host response.

Potent antiangiogenic effects of IL-12 were identified in mid-1990 by the group led by Dr. Judah Folkman [40]. These effects were associated with IFN-γ production, and further on, two more downstream mediators were described: IFN-γ-inducible protein 10 (IP-10, CXCL10) and monokine induced by IFN-γ (MIG, CXCL9) [41]. The importance of IL-12 in controlling tumor-associated angiogenesis has been underscored by a recent observation that antiangiogenic therapy with vascular endothelial growth factor receptor (VEGFR) inhibitors, sunitinib and sorafenib, promoted metastasis of hepatocellular carcinoma model by suppressing host-derived IL-12B (IL-12-p40) [42]. In parallel to the investigations concerning its antiangiogenic actions, the studies on the effects of IL-12 on tumor stroma showed that this cytokine is capable of triggering, partly by IFN-γ, reversion of tumor evasion strategies mediated by myeloid-derived cells within the tumor mass [34], as well as that a collapse of tumor stroma following local secretion of IL-12 can be mediated by Fas [33]. IL-12 was also suggested to alter the expression of endothelial adhesion molecules, such as VCAM-1, that play a role in leukocyte recruitment to the tumor microenvironment [43].

An important characteristic of IL-12, identified in the studies also conducted by our research team, is that it shows a strong tendency to synergize in its biological actions with several other cytokines (reviewed in [44]). Classical examples of such cytokines are TNF-α [45, 46], IL-2 [47, 48], IL-15 [49, 50], IL-18 [50, 51], or GM-CSF [52]. Interesting observations have also been made regarding the actions, either positive or negative, of IL-12 on hematopoiesis [53–55], which can be of importance in cancer patients as well.

In summary, IL-12 possesses multiple biological properties that are capable of governing immune effector actions against a variety of malignancies and, despite some setbacks, remains the center of interest as a recognized anticancer immunotherapeutic agent.

## IL-12: a successful antitumor agent in preclinical studies

In accordance with its ability to stimulate many different direct and indirect antitumor activities belonging to innate immunity, adaptive immunity, and non-immune mechanisms (see above), IL-12 has proven to be very effective in animal models of tumor therapy. This cytokine has been successfully applied in dozens of experimental models in mice involving both solid tumors and hematologic malignancies including poorly immunogenic tumors [56–60]. Many attempts have been made to further potentiate the antitumor effects of IL-12. Antitumor activity of IL-12 can be effectively improved by its combination with various therapeutic modalities: chemotherapeutics, cytokines, antibodies, antiangiogenic agents, radiotherapy, adoptive therapy, and tumor vaccines (Table 1).

**Table 1:** Antitumor effects of interleukin 12 potentiated by various therapeutic modalities in experimental models

Therapeutic modality	Animals	Tumor cells	Refs.
Cyclophosphamide	Mice	MB-49 bladder carcinoma, B16 melanoma	[60]
Paclitaxel	Mice	MmB16 melanoma	[61]
5-fluorouracil	Mice	L1210 leukemia	[62]
5-aza-2′-deoxycitidine	Mice	L1210 leukemia, B16F10 melanoma	[58]
Mitomycin	Rabbits	Hepatocellular carcinoma	[63]
Mitoxantrone	Mice	L1210	[64]
Doxorubicin	Mice	L1210	[65]
Cisplatin	Mice	MmB16	[46]
IL-2	Mice	Mammary carcinoma	[66]
IL-18	Mice	MCA205 fibrosarcoma	[67]
TNF-α	Mice	B16F10 melanoma, Lewis lung carcinoma, L1 sarcoma	[45]
G-CSF	Mice	MmB16 melanoma	[53]
GM-CSF	Mice	MmB16 melanoma	[52]
IFN-α	Mice	B16F10 melanoma	[68, 69]
Cetuximab	Athymic mice	Human head and neck carcinoma	[30]
Vasostatin	Nude mice	CA-46 Burkitt lymphoma, SW620 human colon carcinoma	[70]
Radiotherapy	Mice	Lewis lung carcinoma	[71]
Cytokine-induced killer cells	Mice	DB7 mammary carcinoma	[72]
Vaccine (dendritic cells pulsed with tumor cell lysate)	Mice	B78-H1 melanoma Hepatocellular carcinoma BNL	[73] [74]
Vaccine (IL-2-transduced tumor cells)	Mice	TSA mammary adenocarcinoma SR-B10A glioma	[75] [76]
Peptide vaccine	Mice	Meth A sarcoma	[77]

Chemotherapy is regarded as an established tumor treatment but it can potentially inhibit the development of antitumor immunity interfering with proliferation and/or viability of cells participating in immune response against tumor. Nevertheless, several chemotherapeutics, for example cyclophosphamide, paclitaxel, 5-fluorouracil, 5-aza-2′-deoxycitidine, mitomycin, doxorubicin, and mitoxantrone, have been shown to demonstrate improved antitumor activity in mice when combined with immunotherapy ([65], for review, see [78, 79]). Immunopotentiating effects of chemotherapeutics can include destruction of tumor cells, facilitating the release of tumor-associated antigens and improving the presentation of these antigens by dendritic cells to T cells [80]. Doxorubicin can sensitize tumor cells to cytotoxic activity of T cytotoxic cells and NK cells [81]. Radiotherapy can also activate antitumor immunity [82]. Combining radiotherapy and IL-12 may also have additional advantage as IL-12 could not only potentiate antitumor effects of radiotherapy [71] but could diminish acute radiotherapy injury, according to observation in non-human primates [83]. Potentiated therapeutic effects of IL-12 used in combination with antitumor antibodies [30], as demonstrated in murine xenograft model, are probably caused by increased expression of activating Fcγ receptors, which participate in antibody-dependent cell-mediated cytotoxicity [29, 30].

Although application of IL-12 in mice was found to prevent the development of cancer cachexia [84], it was accompanied by hematologic toxicities including anemia, lymphopenia, neutropenia, and also muscle and hepatic toxicities [85]. In squirrel monkeys, IL-12 produced hypoproteinemia, hypophosphatemia, and hypocalcemia. Enlargement of lymph nodes, splenomegaly, and bone marrow hyperplasia was also observed [86], and these hematologic side effects may be provoked by IFN-γ and TNF-α production stimulated by IL-12 [87]. It is worth mentioning that erythropoietin was found to prevent the development of IL-12-induced anemia and thrombocythemia in mice [54] and G-CSF prevented the suppression of bone marrow myelopoiesis [53]. Unexpectedly, G-CSF was also able to potentiate the antitumor effects of IL-12 in a murine melanoma model [53]. Toxicities induced by the use of IL-12 in experimental animals were originally regarded as not serious enough to postpone the initiation of clinical trials.

To further attenuate IL-12-induced toxicities and potentiate its effectiveness in experimental tumor therapy, various gene therapy protocols have been used, enabling local and prolonged release of this cytokine. The IL-12 gene has been introduced in various viral [88–90] and non-viral [91–93] vectors, directly into growing tumors [92, 94–96] or in IL-12-engineered fibroblasts injected at the site of an established tumor [97]. IL-12 gene has also been successfully used in vaccines consisting of tumor antigens [98], tumor cells [99, 100], or dendritic cells [101–103]. Moreover, IL-12 has strengthened the antitumor activity of adoptive therapy with targeted T cells engineered to secrete IL-12 [104] or therapy with oncolytic *Herpes simplex* virus expressing IL-12 [105]. Examples of application of IL-12 gene therapy combined with other therapeutic approaches in experimental tumor models are presented in Table 2.

**Table 2:** Tumor gene therapy with interleukin 12 potentiated by various therapeutic modalities in experimental models in mice

Therapeutic modality	Tumor cells	IL-12 gene	Refs.
Paclitaxel	B16 melanoma	IL-12 in adenoviral vector; intratumoral injection	[59]
All-*trans*-retinoic acid in liposomes; i.v. injection	Colon 26 adenocarcinoma	IL-12-encoding plasmid in liposomes; distant injection	[106]
Angiostatin; i.d. injection	B16F10 melanoma	IL-12-encoding plasmid; intratumoral injection	[107]
rIL-2; systemic administration	MCA-105 sarcoma, MC-38 adenocarcinoma	IL-12-producing fibroblasts; intratumoral injection	[47]
IL-2 in adenoviral vector; intratumoral injection	Mammary adenocarcinoma	IL-12 in adenoviral vector; intratumoral injection	[108]
rIL-15; local administration	B78-H1 melanoma	IL-12-producing tumor cells; intratumoral injection	[49]
IL-18; distant injection of IL-18-producing tumor cells	SCK mammary carcinoma	IL-12-producing tumor cells; distant injection	[109]
IL-27; distant injection of IL-27-encoding plasmid	CT26 adenocarcinoma 4T1 adenocarcinoma	IL-12-encoding plasmid; distant injection	[110]
IP-10 in adenoviral vector; intratumoral injection	PyMT-induced mammary adenocarcinoma, MCA 207 sarcoma	IL-12 in adenoviral vector; intratumoral injection	[111]
Lymphotactin in adenoviral vector; intratumoral injection	PyMT- or Neu-expressing mammary adenocarcinoma	IL-12 in adenoviral vector; intratumoral injection	[112]
CD80 in adenovirus vector; intratumoral injection	MT1A2 mammary adenocarcinoma	IL-12 in adenovirus vector; intratumoral injection	[113]
Agonistic anti-CD137 agonistic antibody; systemic administration	B16-OVA melanoma, TC-1 lung carcinoma	IL-12 in SFV vector	[114]
CD137 ligand 10 in adenoviral vector; intratumoral injection	MCA26 colon carcinoma	IL-12 in adenoviral vector; intratumoral injection	[115]
CpG ODN; intratumoral injection	B78-H1 melanoma	Vaccine (IL-12-transduced tumor cells)	[116]

Although IL-12 is regarded as one of the most powerful immunostimulatory cytokines, IL-12 gene therapy could still be improved by combination with some other immunotherapeutic modalities employing cytokines, for example, with IL-2 [108] and lymphotactin [112] released by tumor cells following the intratumoral injection of adenoviral vectors and with tumor vaccine expressing IL-18 [109]. In another model, distant injection of IL-12- and IL-27-encoding plasmids resulted in eradication of CT26 tumor in all treated mice [110] and intratumoral injection of IL-12 gene-transduced B78-H1 melanoma cells in combination with IL-15 similarly cured all treated mice [49]. Synergistic interaction of IL-12 gene therapy was also observed with B7.1 (CD80) injected intratumorally in an adenoviral vector [113].

## Clinical trials with IL-12 in cancer immunotherapy: unfulfilled hopes and new trends in IL-12-based approaches

Very encouraging results in preclinical studies and acceptable toxicities in animal models prompted the use of IL-12 in cancer patients in the mid-90s of the last century. Three centers started clinical trials: University of Pittsburgh, PA; Genetics Institute (Cambridge, MA, USA); and Hoffman La Roche (Nutley, NJ, USA). The first group used genetically engineered autologous fibroblasts secreting IL-12, in patients with melanoma or breast cancer. In this small pilot study, IL-12-producing cells were injected peritumorally once a week [117]. Both Genetics Institute and Roche initiated larger clinical trials using recombinant IL-12 but treatment regimens differed in several respects. In the Roche phase I clinical trial, only patients with renal cell carcinoma were recruited and IL-12 was injected subcutaneously once or three times weekly. Genetics Institute applied a “more aggressive” dosing regimen: consecutive intravenous daily injections of IL-12. In the phase I trial, maximal tolerated dose of 500 ng/kg/day was determined. Unexpectedly, this dose was found toxic in phase II trial and severe side effects of the treatment developed in 12 of 17 enrolled patients leading to death of two patients. This resulted in the immediate halting of all trials with IL-12 by the US FDA [118]. Explanation for the different tolerability in phase I versus phase II trial was a change in the dosing schedule. In the phase I trial, a single dose of IL-12 was administered before the multiple-dose regimen. This initial priming dose of IL-12, given to determine the pharmacokinetic profile of the cytokine, was found critical for protection from the severe toxicity [119]. Finally, after several months of suspension, clinical trials were resumed in several centers [120].

Antitumor effects of IL-12 were evaluated in various treatment schedules: intravenous [121–123] versus subcutaneous [124, 125] or even intraperitoneal application [126], daily and five consecutive injections every 3 weeks [121, 122, 127], or at 1 [124, 125, 128], 2 [123], or 3 [129] doses weekly in several-week cycles. Maximal tolerated doses in escalating dose protocols ranged, in relation to the treatment schedule, usually between 250 and 500 ng/kg. In some, more intensive treatment schedules, a priming injection of IL-12 was necessary [129]. What was interesting, pretreatment with a priming dose of IL-12 markedly reduced the toxicity of this cytokine, allowing subsequent administration of relatively high doses, but this regimen did not improve the therapeutic outcome of IL-12. Treatment with IL-12 was associated with systemic flu-like symptoms (fever, chills, fatigue, arthromyalgia, headache) and—more difficult to control—toxic effects on the bone marrow and liver. Hematologic toxicity observed most commonly was neutropenia and thrombocytopenia, and hepatic dysfunction manifested in transient (dose-dependent) increase in transaminases, hyperbilirubinemia, and hypoalbuminemia [119, 121, 122, 124, 129]. Some patients experienced inflammation in mucus membranes (oral mucositis, stomatitis, or colitis) [121]. These toxic effects of IL-12 were related to the secondary production of IFN-γ, TNF-α but also chemokines: IP-10, MIG [123, 128].

Early clinical studies with IL-12, in spite of high expectations, did not yield satisfactory results. Repeated injections of IL-12, after initial stimulation of massive production of IFN-γ, in most patients led to adaptive response and a progressive decline of IL-12-induced IFN-γ concentration in blood [121, 124, 130], attributed partly to negative feedback mechanisms related to overproduction of IL-10 [124, 130]. In fact, as reported by Gollob et al. [123], objective clinical response or stabilization of disease was observed mainly in IL-12-treated cancer patients with sustained production of IFN-γ. This observation was confirmed in the later studies by Bekaii-Saab et al. [131], who showed additionally that the source of continuously produced IFN-γ was CD56^+^ (NK) cells but not T cells. Apart from negative feedback mechanisms, the major reason of marginal efficacy of IL-12 in cancer patients, as opposed to animal tumor models, was probably strong immunosuppressive milieu, typical for tumor microenvironment in humans. In contrast to mice, human tumors seem to consist of much more heterogenous population of tumor cells, developed as a result of tumor escape mechanisms, and contain strongly immunosuppressive soluble and cellular components (including myeloid-derived suppressor cells) that are resistant to IL-12-induced antitumor activity [132, 133].

Limited clinical efficacy of IL-12 used in a monotherapy schedules prompted the investigation of combination treatments. A number of combined approaches were tested in 1995–2005, that is, in the period of the most intensive studies on the antitumor effect of IL-12 in the clinic. The list of these studies is presented in Table 3.

**Table 3:** Summary of clinical studies on the antitumor effects of IL-12-based treatment in combination therapies or gene therapy

Therapeutic modality	Route of IL-12 (or IL-12-based vaccine) administration	Tumor	Refs.
Combined treatment			
Trastuzumab	i.v.	Breast, pancreas, cervical cancer	[134]
Trastuzumab and paclitaxel	i.v. or s.c.	Breast, colon, and other cancers	[131]
Rituximab	s.c.	Non-Hodgkin’s lymphoma	[135]
Peptide vaccine with adjuvant^a^	IL-12 + alum or GM-CSF, s.c. at vaccine injection site	Melanoma	[136]
Peptide vaccine with adjuvant^b^	i.d. at vaccine injection site	Melanoma	[137]
Idiotype vaccine ± GM-CSF	s.c.	Multiple myeloma	[138]
Peptide-loaded PBMCs^c^	s.c. adjacent to immunization site	Melanoma	[139]
Pegylated liposomal doxorubicin	s.c.	AIDS-associated Kaposi sarcoma	[140]
IL-2	i.v.	Melanoma, renal cancer	[44, 141]
IFN-α	i.v. or s.c.	Melanoma, renal cancer, and other cancers	[142, 143]
Gene therapy			
IL-12-transduced autologous fibroblasts	Peritumoral	Melanoma and other cancers	[144]
Adenovirus encoding IL-12	Intratumoral	Liver, colorectal, pancreatic cancer	[145]
Autologous dendritic cells transfected with adenovirus encoding IL-12 gene	Intratumoral	Gastrointestinal carcinomas	[146]
IL-2 gene modified autologous melanoma cells	s.c.	Melanoma	[147]
Canarypox virus expressing IL-12	Intratumoral	Melanoma	[148]
Canarypox virus expressing IL-12 + expressing B7.1	Intratumoral	Melanoma	[149]

^a^Peptides: gp100_209–217_ (210M), MART-1_26–35_ (27L), tyrosinase_368–376_ (370D), adjuvant: Montanide ISA 51

^b^Peptides: gp100_209–217_ (210M), tyrosinase_368–376_ (370D), adjuvant: Montanide ISA 51

^c^Peptide: Melan-A_27–35_

Generally, IL-12—when used either as monotherapy or combined with other agents—with the exception of some studies (see below) did not demonstrate potent sustained therapeutic efficacy. Detailed description of these investigations is beyond the scope of the present article and can be found in earlier comprehensive reviews [37, 44]. Only few IL-12-based clinical trials showed encouraging results and deserve comments: application of IL-12 in patients with cutaneous T-cell lymphoma (CTCL), with non-Hodgkin’s B-cell lymphoma, and with AIDS-associated Kaposi sarcoma.

### Cutaneous T-cell lymphoma (CTCL)

CTCLs are T-cell lymphomas, confined primary to the skin, with the most common variants mycosis fungoides (MF) and, more advanced type, Sézary syndrome. The rationale for testing the efficacy of IL-12 in patients with CTCL was the depressed function of Th1 cells and deficient production of IFN-γ in these patients, the possibility of subcutaneous and intralesion application of IL-12, and a relative susceptibility of this type of neoplasia to immune response-modifying agents [150]. In the study by Rook et al. [151], 10 patients with CTCL, including 3 with Sézary syndrome, were treated with 50, 100, or 300 ng/kg IL-12, subcutaneously or intralesionally twice a week. The treatment was continued for up to 24 weeks. From among nine patients evaluated, only one patient did not respond, and in two patients, complete responses were documented. Skin biopsy specimens from regression lesions showed an increase in cytotoxic CD8^+^ T cells. Adverse effects associated with IL-12 injections were usually mild and short-lived but, what is interesting, one patient experienced mental problems following prolonged therapy (depression) and discontinuation of treatment was necessary.

In another, phase II trial, 23 patients with early-stage MF, who failed at least 3 previous treatments (median 5 prior therapies), were treated subcutaneously at an initial dose of 100 ng/kg IL-12 for 2 weeks and next biweekly with 300 ng/kg IL-12 [152]. Ten patients completed 6 months of treatment and continued therapy for 24 months. What should be stressed, a high rate of response to treatment was achieved (43 % partial response, 30 % minor response, 22 % stable disease) but 52 % of patients ultimately progressed. Some patients initially progressed but, continuing IL-12 injections, achieved minor or even partial responses. Treatment with IL-12 was relatively well tolerated but 5 patients discontinued treatment because of adverse effects. One patient (a 78-year-old man) died of hemolytic anemia, probably exacerbated or even induced by IL-12 therapy [152].

### Hodgkin’s and non-Hodgkin’s lymphoma

The study on the effects of IL-12 on non-Hodgkin’s B-cell lymphoma (NHL) reported by Younes et al. [153] included patients with recurrent or refractory neoplasia (mainly diffuse large cell and follicular—grade I/II). Eleven patients were treated intravenously with 250 ng/kg of IL-12 daily for 5 days every 3 weeks (preceded by an initial test dose of 250 ng/kg), and 21 patients were treated with twice-weekly IL-12 subcutaneous injections at 500 ng/kg (in case of toxicity, the dosage was reduced to 300 ng/kg). Six of 29 evaluable for response patients (21 %) achieved a partial response or complete remission, and 10 patients (34 %) had stable disease. Of note,

- patients with follicular grade I/II lymphoma seemed to respond better than patients with diffuse large cell lymphoma and were characterized by a lower rate of progressive disease (27 vs. 64 %),
- response rates were related to the route of IL-12 administration: intravenous treatment was more effective than subcutaneous injections (partial and complete response 40 vs. 7 %),
- all responding patients had low volume disease (diameter of the largest lesion <3 cm).

In the same study, ten patients with relapsed Hodgkin’s disease were also included [153]. None of these patients achieved a meaningful clinical response but half of them experienced stable disease. However, all the patients were treated subcutaneously, and regarding much better response in NHL patients in intravenous protocol, it cannot be excluded that the lack of effectiveness of IL-12 in these patients resulted from the treatment schedule rather than the type of lymphoma.

The efficacy of IL-12 for the treatment for patients with recurrent B-cell non-Hodgkin’s lymphoma was also tested in combination with rituximab with the hope that due to the strong stimulatory effect of IL-12 on NK cells, this cytokine would potentiate antibody-dependent cell-mediated cytotoxicity (ADCC) of rituximab [154]. A similar treatment schedule to that described above was applied (IL-12 was given s.c. twice weekly at doses 500 and 300 ng/kg or lower). Objective responses (complete or partial) occurred in 69 % patients. The authors also observed a trend toward a higher complete response rate in patients treated with higher doses of IL-12 [154]. These results seem promising but due to a heterogenous group of patients, differing in regard to disease severity, histological types of lymphoma, and prior therapy, no definite conclusion can be drawn as to the real benefit of IL-12 in the combination schedule. In fact, response rates in NHLs in a monotherapy with rituximab, given at the same dose (375 mg/m^2^) and similar schedule, has been found to range between 47 and 73 % [155].

### Kaposi sarcoma

Kaposi sarcoma (KS) is a lymphangioproliferative disease caused by Kaposi sarcoma-associated herpes virus (KSHV), also known as human herpesvirus 8 (HHV-8). Taking into account the stimulation of production of antiangiogenic chemokines by IL-12, its role in the promotion of cell-mediated immune response, and augmentation of NK activity by this cytokine (see “Biological effects of IL-12 and its role in the antitumor defense mechanisms” section of this review), there was a strong rationale to use IL-12 in patients with AIDS-associated Kaposi sarcoma. In a dose-escalating study by Little et al. [156], patients with AIDS-associated KS were treated with IL-12 at doses 100 up to 750 ng/kg twice weekly. In accordance with other studies, the dose 100 ng/kg was found ineffective and 500 ng/kg was established as the maximal tolerated dose. Of 24 evaluable patients treated with higher doses, 17 had partial or complete response (71 %). What should be stressed, responses occurred after continued IL-12 therapy (median time to response: 18 weeks), and complete regression of the tumor in some patients occurred as late as at 243 or 253 weeks after entering the study. Patients with less advanced disease responded better than high risk patients. Of note, apart from the typical side effects associated with IL-12 therapy (flu-like symptoms, hepatotoxicity, suppression of bone marrow function), psychoneurological problems appeared in some patients: mood worsening and depression. Based on the results of studies in numerous animal models showing synergistic antitumor effects of IL-12 with chemotherapy (see “IL-12: a successful antitumor agent in preclinical studies” section), phase II trial was initiated in which patients with advanced AIDS-associated KS were treated with pegylated liposomal doxorubicin and IL-12 for 18 weeks and next with IL-12 alone [140]. In the combination therapy, IL-12 was injected subcutaneously twice weekly at a dose of 300 ng/kg and in the maintenance phase, the patients were treated with 500 ng/kg. Like in the previous study [156], the patients received independently highly active antiretroviral therapy (HAART). The majority of patients experienced objective responses (83 %), including 25 % complete responses. These encouraging results were by no means related to HAART alone, and the major contributing factor was certainly doxorubicin-based cytotoxic chemotherapy. However, the addition of IL-12 could be beneficial since a substantial number of responses occurred in the maintenance phase. Anyway, randomized trials are needed to assess the exact extent of IL-12 benefits.

### Current trends

**Fig. 2:**
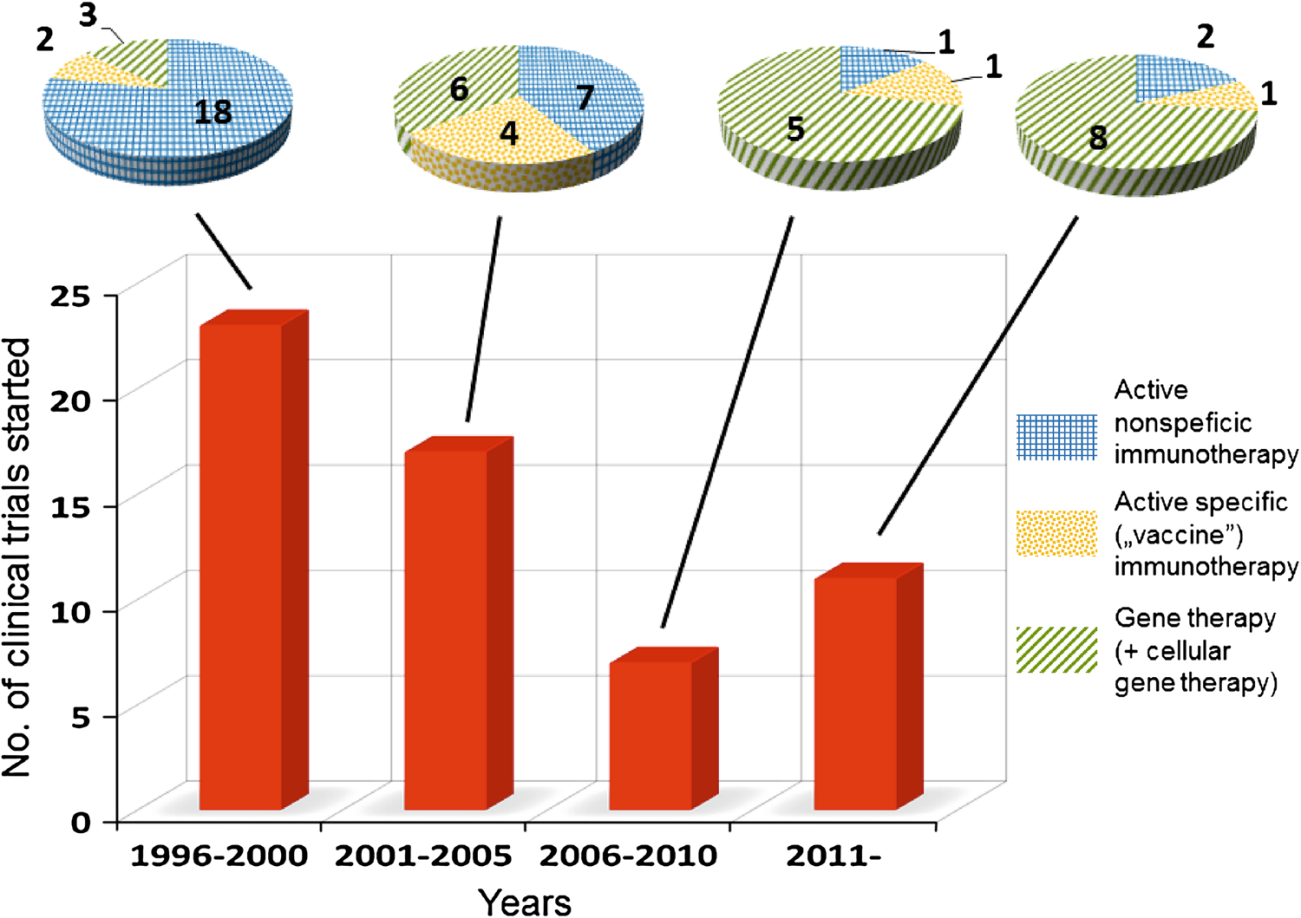
Number of IL-12-based clinical trials in the field of tumor immunotherapy that have been started since 1996 and have been registered in ClinicalTrials.gov database (http://www.clinicaltrials.gov). The histogram comprises studies in which IL-12 was used alone or in combination, either as a main therapeutic or administered in an adjuvant setting including gene therapy

On the basis of the largest registry of clinical studies in the world (http://www.clinicaltrials.gov), 58 clinical trials testing IL-12-based therapy, predominantly located in the United States, for the treatment for patients with various types of cancers have been started or completed (key words for survey: IL-12, interleukin 12, tumor, cancer) so far. Analyzing the history of these trials, three stages of interest in the application of IL-12 in clinical oncology can be distinguished. Years 1996–2005 was a period of most intensive studies, aimed at establishing maximal tolerated doses of IL-12, optimal treatment schedule, and the most susceptible tumors. As described in the previous section, IL-12 was characterized by a very narrow therapeutic index. In fact, only few studies reported promising results with sporadic overt tumor regressions (apart from patients with AIDS-associated KS). Due to the low response to IL-12 and its high toxicity, accrual to some trials was even stopped [157]. However, after a 5-year discouragement (2006–2010), it seems that the interest in IL-12 therapeutic potential has revived but the strategy of its use is being revised. Generally, IL-12-based therapies can be divided into three categories: (1) active non-specific immunotherapy, aimed at activation of predominantly innate mechanisms of antitumor response, e.g., application of IL-12 alone or in combination with chemotherapy or monoclonal antibodies; (2) active specific (“vaccine”) approach, directed to the stimulation of adaptive antitumor response mainly, e.g., using IL-12 as an adjuvant with tumor cells or tumor antigen-derived peptides; and (3) gene therapy, including cellular adoptive treatment (Fig. 2). While the first approach predominated in years 1996–2005, most recently initiated clinical trials have been concentrated on gene therapy (Fig. 2). In addition, in recent trials, regarding high toxicity of IL-12, most IL-12-based therapies are restricted to intratumoral/local treatment. The rational for this approach is not only avoiding toxic effects. As shown and stressed in many recent reports, strong immunosuppressive mechanisms operate in the microenvironment in advanced tumor and IL-12 is expected, on the one hand, to overcome this phenomenon and, on the other hand, to induce specific antitumor mechanisms [158, 159].

At present, more than ten IL-12-based clinical trials in cancer patients are ongoing (as of September 4, 2013). Six trends are worth discussing.

#### Intraperitoneal administration of IL-12 plasmid formulated with a synthetic polyethyleneglycol–polyethyleneimine–cholesterol (PPC) lipopolymer (EGEN-001, phIL-12/PPC)

These gene therapy studies were initiated in 2005, and till 2013, six clinical trials have been started, according to the ClinicalTrials.gov database. In the studies, a gene-based IL-12 therapeutic, named EGEN-001, is injected intraperitoneally as an alternative to recombinant IL-12 delivery. EGEN-001 consists of 100–150 nm nanoparticles containing a human plasmid (phIL-12) that encodes functional IL-12 protein and a synthetic DNA-delivery system comprising polyethyleneglycol–polyethyleneimine–cholesterol (PPC). The DNA-delivery system was designed to improve IL-12 gene transfer by blocking rapid degradation of the plasmid and facilitating its uptake by target cells. Of note, there were earlier trials with direct intraperitoneal administration of recombinant IL-12 in patients with peritoneal carcinomatosis but satisfactory effects were not achieved [160].

Results of the phase I, dose-escalating study investigating the safety, tolerability, and preliminary efficacy of phIL-12/PPC in women with recurrent ovarian cancer were published in 2010 [161]. In the study, 13 patients were recruited. Repeated intraperitoneal administration of phIL-12/PPC for 4 weeks (weekly infusions of doses: 0.6, 3, 12, or 24 mg/m^2^) was well tolerated and induced mild-to-moderate side effects, most frequently abdominal pain and discomfort. In six patients, a decrease in CA-125 level or stabilization of this cancer antigen was observed. There was an overall clinical response of 31 % stable disease and 69 % progressive disease as assessed 4–6 weeks post-treatment. The overall survival of patients treated with low doses was 12.7 months and did not differ from that of historical control but administration of higher doses seemed to prolong survival and resulted in the mean survival time of 23 months. However, these encouraging data must be interpreted with caution not only due to the small sample size and possible selection bias but also because of additional treatment (chemotherapy) of some patients [161].

In a parallel study, patients with recurrent platinum-sensitive ovarian cancer were treated with phIL-12/PPC in combination with carboplatin and docetaxel. The patients were intraperitoneally infused with escalating doses of phIL-12/PPC (12, 18, or 24 mg/m^2^) once every 10–11 days for four treatments or, in additional 24 mg/m^2^ group, up to eight treatments. Docetaxel and carboplatin were given at 3-week intervals for up to 6 cycles. Seventeen percent of the patients had complete response, 33 % partial response, 42 % stable disease, and 8 % of the patients progressed [162].

The above-described studies prompted the initiation of phase II trials with phIL-12/PPC in combination with

- pegylated liposomal doxorubicin in patients with epithelial ovarian cancer, fallopian tube cancer, and primary peritoneal cavity cancer (NCT01118052, started in 2010; NCT01673477, started in 2012; and NCT01489371, started in 2012),
- standard chemotherapy in patients with colorectal peritoneal carcinomatosis (NCT01300858, started in 2011).

#### Intratumoral delivery of IL-12 plasmid by electroporation

In preclinical models of direct intratumoral injections of IL-12 gene-containing plasmids, this therapeutic approach was found effective not only in inducing systemic antitumor response but also in tumor regression (Table 2). However, in clinical trials, results were mostly disappointing [163, 164], related, among others, to low gene transfer efficiency. To overcome this problem, a phase I dose-escalation trial was started in 2004 using intralesion IL-12 plasmid injections accompanied with electroporation performed at the site of plasmid administration (NCT00323206). The trial recruited 24 patients with documented metastatic melanoma, divided into 3 or 6 persons groups injected with a total dose up to 3.8 or 5.8 mg of plasmid per treatment [165]. Under local anesthesia and intravenous analgesic medications, an applicator containing six needle electrodes arranged in a circle was inserted into the tumor and six pulses (1,300 V/cm) lasting 100 μs were applied using a Medpulser DNA EPT System Generator. The procedure was associated with minimal systemic toxicity—induced only transient pain—and was repeated three times (on day 1, 5, and 8) leading to significant in situ production of IL-12 (up to 2,813 pg/g of tumor mass). Biopsies of injected lesions showed necrotic areas and lymphocyte infiltrations. In 53 % patients, there was evidence of systemic response and three patients experienced complete response (of note, one of these patients was additionally treated with dacarbazine) [165].

These promising results encouraged to continue the study or to explore intratumoral pIL-12 injections and in vivo electroporation in other types of cancer:

- in patients with Merkel cell cancer (NCT01440816, initiated in 2011),
- in advanced-stage cutaneous and *in transit* malignant melanoma (NCT01502293, a multicenter phase II study, started in 2011),
- in patients with cutaneous T-cell lymphoma (mycosis fungoides and Sézary syndrome) (NCT01579318, phase II trial, started in 2012).

Enrollment to these clinical trials is currently underway (as of September 4, 2013).

#### Adoptive immunotherapy with IL-12-engineered lymphoid cells

Adoptive immunotherapy is based on the isolation, ex vivo expansion (activation), and reinfusion of immune cells, predominantly lymphoid cells, into a tumor-bearing patient. In first trials with lymphokine-activated killer (LAK) cells and tumor-infiltrating lymphocytes (TILs) in second half of eighties of the last century, despite very promising results in animal models, this therapy was found weakly effective. The major limitation was a short half-time of circulation of the infused cells and their poor homing to the tumor (for review, see [166]). Over time, much progress has been made in this field [167], due to

- optimization of the treatment protocol (e.g., introducing lymphodepleting chemotherapy),
- selection of appropriate subpopulations to be administered,
- genetic modifications of the cells.

Recently, two clinical trials have been started with genetically modified, IL-12-secreting lymphocytes. Both studies have been aimed at treatment of melanoma patients and have been coordinated by Rosenberg and colleagues. In one of these studies (phase I/II) (NCT01236573, started in 2010), patients with metastatic melanoma receive a non-ablative lymphocyte-depleting preparative regimen followed by infusion of CD8^+^-enriched, genetically modified and ex vivo expanded, tumor-infiltrating lymphocytes (TILs). The cells (isolated from metastatic deposits) are transduced with retroviral vector containing an inducible single chain IL-12 gene driven by an NFAT responsive promoter. Such a genetic modification enables the secretion of IL-12 by the cells following specific antigen recognition via T-cell receptor. The investigators anticipate that, like in animal models, IL-12 produced locally by CD8^+^ T cells will trigger acute inflammation and will induce expression of Fas within tumor-infiltrating macrophages, dendritic cells, and myeloid-derived suppressor cells (MDSC) leading to reversion of dysfunctional antigen presentation in the microenvironment and to eradication of the tumor [33]. However, a very recent report [168] has suggested superiority of unselected TILs versus CD8^+^-enriched TILs in adoptive cell therapy, so the investigators consider the option of using TILs without CD8^+^ T-cell enrichment in some patients.

The other clinical trial based on adoptive transfer of cells has been planned to treat metastatic melanoma patients with a non-myeloablative lymphocyte-depleting regimen followed by the administration of gene-engineered lymphocytes co-transduced with genes encoding IL-12 and T-cell receptor specific for NY-ESO-1 tumor antigen (NCT01457131, started in 2011). However, several weeks after the start of the trial, the study suspended participant recruitment and—till now—has not been resumed.

#### Intratumoral injections of IL-12-expressing adenovirus vector in combination with oral activator ligand

In order to maximize the safety profile of IL-12-based therapy by reducing systemic expression to this cytokine, an approach has been invented in which the expression of IL-12 can be regulated. In this approach, patients are injected intratumorally with Ad-RTS-hIL-12—an adenoviral vector engineered for controlled expression of IL-12 with RheoSwitch Therapeutic System^®^ (RTS^®^) technology. Injection of the vector leads to local production of two unstable heterodimeric receptor proteins with potential binding to an inducible promoter regulating the production of IL-12. Small molecule activator ligand (INXN-1001), when administered orally, induces stable conformation of the heterodimeric proteins enabling the productive interaction of this complex with the promoter and initiating the expression of IL-12.

The safety and tolerability of Ad-RTS-IL-12 with INXN-1001 is currently being tested in a phase I/II study (NCT01397708, started in 2011) in patients with advanced-stage III or IV melanoma. Preliminary results are encouraging: clinical responses were observed in five of seven patients treated with Ad-RTS-IL-12 and high doses of INXN-1001 (100 or 160 mg/day). The responses were associated with intratumoral IL-12 mRNA expression and were reflected by a decrease in size of both injected and distant lesions. One of the patients treated at the 160 mg/day dose of INXN-1001 had stable disease for 20 weeks [169].

Ad-RTS-IL-12-based therapy has also been studied in a phase II randomized trial, as a monotherapy or in combination with palifosfamide-tris in patients with recurrent/metastatic breast cancer (ATI001-101, http://mccrc.clinsite.com).

#### Combination of IL-12 with a vaccine containing tumor cells fused with dendritic cells

Immunotherapy with dendritic cells/tumor cells (DC/TC) fusion vaccine represents a promising approach in clinical oncology since it optimizes the presentation of a broad array of tumor antigens along both MHC class I and II pathways [170]. This adoptive immunotherapy, using either autologous or allogeneic dendritic cells, was exploited in the past in patients with different tumors [170–172]). Recently, DC/TC fusions have been tested in patients with multiple myeloma patients. The therapy induced specific antitumor response and disease stabilization in the majority of patients [173] and, when applied following autologous stem cell transplantation, resulted in the marked development of myeloma-specific T cells and eradication of post-transplant residual disease in some patients [174]. In 2009, the same research group, which reported the above-mentioned investigations, started testing the safety of dendritic cell/tumor cell fusion vaccine given with IL-12 for patients with breast cancer (NCT00622401). In the study, a group of patients is vaccinated with cell fusions alone while in other groups, patients are treated additionally with low doses of IL-12 (30 or 100 ng/kg) administered subcutaneously. Investigators expect that, like in experimental animal models, supplementation of IL-12 will promote, on the one hand, antitumor Th1 polarization and, on the other hand, will attenuate immunosuppressive activity of Treg cells [175, 176]. Some data suggest that the therapeutic effects of the combination treatment with IL-12 and DC/TC hybrids may be superior to the hybrids alone, at least in patients with glioma [177]. There is a concern, however, that such a treatment can induce systemic autoimmune response [178]. Of note, sipuleucel-T (a vaccine based on peripheral blood mononuclear cells, including antigen-presenting cells, incubated with prostatic acid phosphatase fused to GM-CSF) was approved in 2010 by the US FDA for the treatment for prostate cancer [179], and individualized dendritic cell-based therapy, consisting of dendritic cells loaded with tumor proteins and stimulated to secrete IL-12, was found beneficial when used in combination with a standard therapy in patients with glioblastoma multiforme [180].

#### Tumor targeting by IL-12-based immunocytokine

One of the approaches aimed at the reduction in toxicity associated with systemic administration of cytokines, including IL-12, is selective targeting delivery by their conjugation with tumor antigen-specific monoclonal antibody. Such biotherapeutics, called immunocytokines, tend to accumulate in the tumor tissue and, by releasing cytokines, directly kill tumor cells or induce a strong inflammatory process eliciting antitumor response [181].

In a phase I study by Rudman et al. [182], an immunocytokine AS1409 targeting extra-domain B (ED-B) fibronectin isoform was used to deliver IL-12 into tumor mass in a small group of 13 patients with melanoma and renal cell carcinoma. ED-B fibronectin is a marker of angiogenesis and is highly expressed in tumor blood vessels and stroma. In general, the immunocytokine was well tolerated but its efficacy was limited. Overall, one patient experienced a partial response but stabilization of the disease was observed in a group of a further five patients.

In 2011, a phase I trial was started with another IL-12-based immunocytokine NHS-IL12 to determine the dose-limiting toxicities and MTD in patients with metastatic or locally advanced tumors (NCT01417546). NHS-IL12 is a fusion protein consisting of two molecules of IL-12 linked to one molecule of humanized monoclonal antibody NHS76. The protein targets necrotic portions of tumor due to its high affinity to single- and double-stranded DNA. Since the study plans to enroll up to 78 patients, there is hope that the results of this trial will be more conclusive than those described in the previous study.

## Conclusions and perspectives

Recent investigations in animal models and in patients with disseminated cancer unequivocally show that the major reason of failure of immunotherapy is the immunosuppressive microenvironment and tumor escape mechanisms operating in the tumor tissue [132, 133]. These mechanisms include both cellular and soluble components and are potent enough to limit the development of durable antitumor response in patients treated with IL-12, despite induction of specific immunity against tumor-associated antigens in some patients [183]. The logical consequence of these setbacks was the commencement of trials of local/intratumoral application of IL-12, including gene therapy and optimizing active specific (vaccine) immunotherapy in which IL-12 was used as an adjuvant. The important step forward has been designing therapeutic protocols enabling controlled in situ expression of IL-12, e.g., based on RheSwitch Therapeutic System^®^ technology (see the previous chapter). Another therapeutic approach awaiting optimization in animal models and resuming in clinical trials is targeting IL-12 to the tumor environment and controlling its local production using chimeric antigen receptor (CAR)-modified T cells engineered with IL-12 gene [184]. The CAR-modified T cells specific for CD19 have recently shown promise in the treatment for acute leukemia and chronic lymphocytic leukemia [185, 186]. In light of the optimistic results of the above-mentioned clinical studies, supported by promising results in improved IL-12-based preclinical models of tumor immunotherapy, there is hope that IL-12 will join (ultimately!) the armamentarium of anticancer agents and selected groups of patients will benefit from the treatment. IL-12-based immunotherapy could be especially efficacious in cancer patients with inherited defects of IL-12 production [187] or with downregulated expression of IL-12 [188].
